# The prognostic value of location and size change of pathological lymph nodes evaluated on CT-scan following radiotherapy in head and neck cancer

**DOI:** 10.1186/s40644-017-0111-y

**Published:** 2017-02-28

**Authors:** Daan Nevens, Olivier Vantomme, Annouschka Laenen, Robert Hermans, Sandra Nuyts

**Affiliations:** 1 Department of Radiation Oncology, University Hospitals of Leuven, Herestraat 49, 3000 Leuven, Belgium; 2 Leuven Biostatistics and Statistical Bioinformatics Centre, University of Leuven, Leuven, Belgium; 3 Radiology Department, University Hospitals of Leuven, Leuven, Belgium

**Keywords:** Cancer of the head and neck, Computed tomography, Neoplasm recurrence locoregional, Radiotherapy, Lymph nodes

## Abstract

**Background:**

Overall survival after chemo-radiotherapy (CRT) for head and neck cancer ranges between 50 and 60% after 5 year of follow-up. Local and/or regional recurrence is the most frequent form of therapy failure. The aim of this study is to investigate whether the initial location and size change of pathological lymph nodes as evaluated on Computed Tomography (CT) studies can help predict outcome.

**Methods:**

One hundred eighty-three patients with lymph node-positive head and neck cancer were treated with radiotherapy (RT) or CRT. CT studies pre- and post-treatment were reviewed for lymph node size and location. Data were correlated with local control, regional control, metastasis free survival, disease free survival and overall survival.

**Results:**

Regarding the risk for distant metastasis, a significant influence was seen for the location of the pathological lymph nodes. The metastatic risk increases when levels IV-V are affected rather than levels I-III. A similar observation is seen for levels VI-VII. Regional control improves with decreasing lymph node diameter and volume as evaluated on CT.

**Conclusions:**

Both location and size change of pathological lymph nodes are of prognostic value after CRT for head and neck cancer.

## Background

Head and neck cancer encompasses a large number of tumor entities originating from subsites such as the nasal cavity, nasopharynx, oral cavity, oropharynx, larynx, hypopharynx and salivary glands. The majority of these tumors are squamous cell carcinoma [1]. Head and neck cancer is the fifth most common cancer worldwide. In the United States about 55,070 new cases were estimated for the year 2014 [2].

Approximately 60 to 80% of patients present with locoregionally advanced disease at time of diagnosis. Concurrent radiation therapy (RT) and chemotherapy has become the standard of care for this subset of patients [3, 4]. Overall survival after chemo-radiotherapy (CRT) ranges between 50 and 60% after 5 year of follow up. Local and/or regional recurrence is the most frequent form of therapy failure after CRT, while failure due to metastasis is much less common [5–7]. Close follow-up of the neck after CRT in this patient group is therefore very important. There is a wide variability regarding the decision for salvage neck dissection as well as post-treatment surveillance imaging.

The aim of this retrospective study is to investigate whether the initial location of the pathological lymph nodes and size changes after treatment evaluated using CT studies, in patients diagnosed with a locoregionally advanced invasive head and neck squamous cell carcinoma, can help predict outcome and can help us in selecting patients who might benefit from a closer follow-up with more frequent radiological examinations.

## Methods

### Patients

Between January 2002 and December 2012, 539 patients with invasive head and neck squamous cell carcinoma were treated with (C)RT at our centre. After the exclusion of patients with in situ cancer, metastasized disease or N0 disease at time of diagnosis, unknown primary tumors, nasopharyngeal or sinonasal primaries, RT at another centre and neoadjuvant chemotherapy or upfront surgery before RT, we had a cohort of 183 patients for further analysis. Of these patients 183 patients, 168 had a CT evaluation 4 months following the end of treatment.

All patients received external beam radiotherapy and were CT planned. The primary tumor and pathological lymph nodes were irradiated to doses ranging from 69.96 to 72 Gy. Mean overall treatment time was 43.4 days (range, 37-58 days). Most patients received concurrent cisplatin. A smaller group was treated with cetuximab or carboplatin/5-fluoro-uracil, or was included in a clinical trial and received alternative or additional therapeutic regimens. Patient and treatment characteristics are presented into more detail in Table 1. For all patients with oropharyngeal tumors, formalin-fixed, paraffin-embedded tissue was centralized (University Hospital of Leuven) for Human papilloma virus (HPV)-status determination. HPV testing was performed using a previously validated algorithm using p16 immunohistochemistry followed by HPV-polymerase chain reaction (PCR). A tumor was regarded as HPV related when both p16 immunohistochemistry as well as HPV-PCR were positive. For p16 immunohistochemistry a purified mouse anti-human p16 antibody (G175-405, BD Pharmigen) was used. Sections were scored as p16 positive when clear p16 immunoreactivity was seen in at least 50% of cells.

**Table 1:** Patient and Treatment Characteristics

Characteristic	No. of patients (*n* = 183)	%
Mean age (y)	59	
Sex		
Male	155	85
Primary tumor site		
Oral cavity	30	16
Oropharynx HPV + HPV – Unknown HPV status	122 20 92 10	67
Hypopharynx	25	14
Larynx	6	3
T classification^a^		
T1	8	4
T2	47	26
T3	51	28
T4	76	42
N classification		
1	32	17
2	142	78
2a	7	4
2b	63	34
2c	72	39
3	9	5
Radiation therapy		
3D CRT	84	46
IMRT	99	54
Systemic therapy		
none	35	19
cisplatin	118	64
carboplatin/5-FU	2	1
cetuximab	11	6
panitumumab	8	4
cisplatin + tirapazamine	8	4
cisplatin + zalutumumab	1	1

*Abbreviations*: *3D CRT* three-dimensional conformal radiation therapy, *IMRT* intensity-modulated radiation therapy, *5-FU* 5-fluoro-uracil, *HPV* Human papilloma virus. ^a^1 patient had 4 synchronous head and neck malignancies and was not included in the T-classification statistics

CT studies were carried out before treatment and 4 months after the completion of treatment. If the CT scan was negative at 4 months, further follow-up was done by clinical examination. PET-CT examination was not routinely performed in the patients.

### Analysis of the CT imaging data

CT-scans prior and following CRT were reviewed for all included patients. All CT studies were performed using multidetector spiral CT. An iodinated contrast agent was injected in most patients; 100 ml at a rate of 1-1.5 ml/s. Scanning was started 80-100 s after the start of the contrast agent injection. The native CT-images were acquired with a slice thickness of 0.6-0.75 mm, and reformatted for display with a slice thickness of 3 mm.

To obtain volumes and diameters of the lymph nodes, every lymph node was contoured on each slide. Consecutively, volumes and diameters were calculated using the Impax Volume Viewing 3D software from Agfa Healthcare. At patient level volumes were obtained by considering the sum of all pathological lymph nodes volumes in one patient. At nodal level individual pathological lymph nodes volumes were used. Lymph node levels were clustered (level I-III, IV-V and VI-VII) and compared pairwise. Furthermore, necrosis, calcifications and ECS was evaluated on all the CT studies. CT criteria for ECS were apparent fat and soft tissue infiltration or infiltration of sternocleidomastoid muscle, internal jugular vein or carotid artery.

All CT scans were analyzed by a single observer (a professor in radiology specialized in head and neck imaging) concerning the presence of pathological lymph nodes, the level of the lymph nodes, and the presence of extracapsular spread or necrosis. The volume and diameter of the lymph nodes were measured by a resident in radiation oncology under the supervision of a radiologist and radiation oncologist specialized in head and neck cancer.

### Statistical analysis

Description of the time-to-event outcome (LC (local control), RC (regional control), MFS (metastasis free survival), DFS (disease free survival), OS (overall survival)) of the patient cohort was based on Kaplan-Meier estimates. The predictive power of localization and volume changes were analyzed by Cox regression models, and results presented as hazard ratios (HR) with 95% confidence intervals. Predictors for binary outcome were analyzed by logistic regression models, and results presented as odds ratios (OR) with 95% confidence intervals. Furthermore, a multivariate analysis was done to correct for T stage, N stage, pre-RT diameter, the presence of necrosis or calcifications within the pathological lymph nodes, HPV and ECS.

Analyses at lymph node-level were based on generalized estimating equations (GEE) to account for clustering of lymph nodes within patients. Size changes of pathological lymph nodes are presented as percentage of change. A negative % indicates decrease, a positive % increase. Disappearing pathological lymph nodes after treatment were considered as a 100% decrease of volume and diameter. Analyses have been performed using SAS software (version 9.4 of the SAS System for Windows).

Volume change was analyzed at patient level as well at individual nodal level. Data at patient level were correlated with RC, MFS, DFS and OS, with inclusion of disappearing pathological lymph nodes (100% decrease of volume) after treatment. At individual nodal level data were correlated with individual lymph node relapse (LNR), and analyzed both with in- and exclusion of disappearing pathological lymph nodes.

Change of largest axial diameter was analyzed on individual nodal level only, both with and without disappearing pathological lymph nodes after treatment.

## Results

### Survival, disease control, CT characteristics

We report the following 2-year outcome rates: LC 86% (95% Confidence Interval: 80-90%), RC 83% (95% Confidence Interval: 77-88%), MFS 77% (95% Confidence Interval: 70-83%), DFS 65% (95% Confidence Interval: 57-71%) and OS 70% (95% Confidence Interval: 63-77%). Median observed follow-up was 5.04 years (Q1 3.48 years; Q3 7.04 years). For 116 patients we had at least 2 years of follow-up.

The pre- and post-CT study characteristics are presented in Table 2 a and b.

**Table 2:** CT parameters on pre and post RT CT study

Variable	Statistic	All
a.		
Sum-volume	*N*	183
	Mean	18.4
	Std	31.21
	Median	9.7
	IQR	(3.9; 19.9)
	Range	(0.3; 257.3)
Largest diameter	*N*	183
	Mean	26.0
	Std	14.46
	Median	23.0
	IQR	(17.0; 32.8)
	Range	(2.6; 123.0)
ECS		
No	n/N (%)	146/183 (79.78%)
Yes	n/N (%)	37/183 (20.22%)
Necrosis		
No	n/N (%)	60/183 (32.79%)
Yes	n/N (%)	123/183 (67.21%)
Calcifications		
No	n/N (%)	180/183 (98.36%)
Yes	n/N (%)	3/183 (1.64%)
b.		
Sum-volume	*N*	168
	Mean	3.8
	Std	8.02
	Median	1.3
	IQR	(0.7; 3.1)
	Range	(0.1; 56.7)
Largest diameter	*N*	168
	Mean	15.2
	Std	9.19
	Median	12.5
	IQR	(9.6; 16.8)
	Range	(5.5; 55.8)
ECS		
No	n/N (%)	150/168 (89.29%)
Yes	n/N (%)	18/168 (10.71%)
Necrosis		
No	n/N (%)	115/168 (68.45%)
Yes	n/N (%)	53/168 (31.55%)
Calcifications		
No	n/N (%)	147/168 (87.50%)
Yes	n/N (%)	21/168 (12.50%)

### Location

**Fig. 1:**
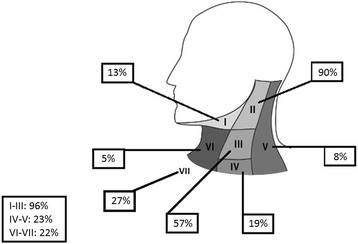
Distribution of affected lymph node levels

The distribution of affected lymph node levels in our patient cohort is presented in Fig. 1. Level II was most frequently affected. Regarding the risk for distant metastasis, a significant influence was seen for the location of the pathological lymph nodes. The metastatic risk increases when levels IV-V are affected rather than levels I-III. A similar observation is seen for levels VI-VII (Table 3). On the other hand, no significant differences were found for RC or DFS.

**Table 3:** Impact of PLN localization on metastatic risk

Levels	Levels	Hazard ratio (95% CI)	*p*-value univariate analysis	*p*-value multivariate analysis^a^
I-III	IV-V	0.325 (0.165;0.639)	0.0011	0.0002
IV-V	VI-VII	0.461 (0.127;0.745)	0.0852	0.0302

Hazard Ratio <1 (>1) means lower (higher) risk for lower levels

*Abbreviations*: *CI* confidence interval, *PLN* pathological lymph node. ^a^Corrected for T stage, N stage, pre-radiotherapy diameter, necrosis, calcifications, extra-capsular spread and HPV

### Size change

#### Volume

At a patient level, following multivariate analysis, less decrease of total lymph node volume on a CT study was significantly associated with a higher risk for regional relapse (OR 1.039, *p* = 0.003), distant metastasis (HR 1.006, *p* = 0.0277) and poorer DFS (HR 1.01, *p* = <0.001) and OS (HR 1.006, *p* = 0.0037). At nodal level, less decrease of volume of an individual lymph node was associated with a higher risk for LNR, both with in- (OR 1.015, *p* = 0.0126) and exclusion (OR 1.009, *p* = 0.033) of completely resoluting pathological lymph nodes.

#### Largest axial diameter

**Fig. 2:**
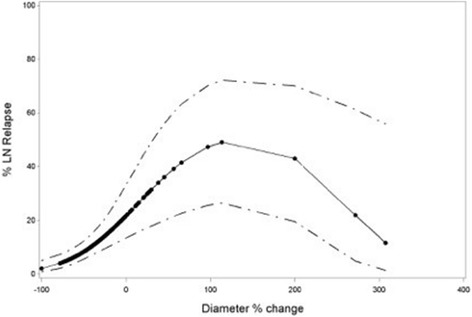
Nonlinear trend of diameter change in relation with risk of Lymph Node Relapse (LN Relapse) with inclusion of completely responding LN’s. The *dots* represent predictions for individual LN’s

**Fig. 3:**
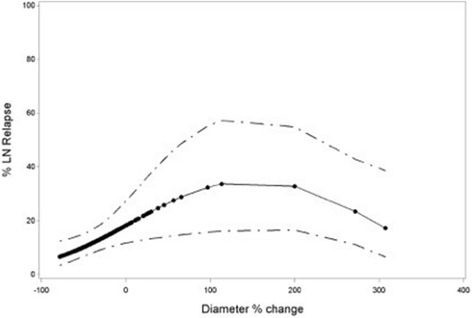
Nonlinear trend of diameter change of diameter change in relation with risk of Lymph Node Relapse (LN Relapse) with exclusion of completely responding LN’s. The *dots* represent predictions for individual LN’s

Less decrease of largest axial diameter or an increase of diameter was correlated with a higher risk for LNR. Both with and without considering completely resoluting pathological lymph nodes, a nonlinear trend was observed for this relation (Figs. 2 and 3). Hence, the OR associated with a 1% increase in diameter around the median value was conducted (Table 4).

**Table 4:** Predictive value of diameter change for lymph node relapse

	Dmax	Odds ratio (95% CI)	*p*-value*
with CR PLN	ΔDmax (nonlinear trend)		<0.001
	ΔDmax (+1% ~ median)	1.012 (1.006;1.017)	<0.001
without CR PLN	ΔDmax (nonlinear trend)		0.0167
	ΔDmax (+1% ~ median)	1.009 (1.003;1.015)	0.0062

OR > (<1) means higher (lower) risk for higher % of change

*Abbreviations*: *LNR* lymph node relapse, *CR PLN* completely resoluting pathological lymph node, *ΔDmax* change in largest axial diameter, *CI* confidence interval. *Corrected for, pre-radiotherapy diameter, necrosis, calcifications and extra-capsular spread

## Discussion

In an era where the role of planned neck dissection after CRT for locoregionally advanced head and neck cancer is diminishing [8–10], using prognostic information derived from the clinic and radiological examinations which can help predict treatment outcome and thereby the necessity of neck dissection, is gaining importance. The goal of this study is to situate the prognostic value of the location of pathological lymph nodes, as well as size change of pathological lymph nodes as evaluated on CT studies in the follow up after (C)RT. Data were conducted from a large patient cohort treated at our institute with (C)RT but without adjuvant neck dissection. As a result of this, data were correlated with clinical outcome, and not with pathology results from neck dissection. Therefore, we made sure to have sufficient follow-up for every patient. Multivariate analysis were done to correct for T stage, N stage, pre-RT diameter, necrosis, calcifications, HPV and ECS, since these factors are known to impact outcome [11–16].

To investigate the prognostic impact of the location of pathological lymph nodes, we analyzed the distribution of affected lymph node levels in our patient cohort, clustered adjacent levels and compared them to each other. As described for overall survival by Jones et al. in a large cohort of 947 patients, one would expect a worse prognosis with pathological involvement of higher numerical lymph node levels [11]. We could establish a significant correlation between location and metastatic risk, without significant impact on the DFS. Involvement of level IV-V entails a higher risk to develop metastasis compared to level I-III. A similar, correlation was seen for involvement of level VI-VII compared to level IV-V; with a higher risk metastatic for involvement of level VI-VII. Presence of retropharyngeal lymph node metastasis (level VII), is known to be a risk factor for both regional and distant recurrence [12–15]. We could validate this result in our patient group for distant recurrence. Based on our data, and previous publications on this subject, we can state that involvement of higher numerical lymph node levels as evaluated on CT studies pretreatment entails a higher metastatic risk and represents more aggressive and advanced disease.

A second goal was to investigate the prognostic value of size change as evaluated on CT studies of pathological lymph nodes after (C)RT (radiotherapy with or without chemotherapy). Therefore we considered both volume and largest axial diameter change. The latter only at individual nodal level, given the fact that after treatment, determination of the largest axial diameter does not necessarily involve the same lymph node. Volume change was both at patient and individual nodal level of significant prognostic value. The less pronounced the volume reduction, the poorer the RC, DFS, MFS, OS and, at individual nodal level, and the more LNR. Due to linear relationships between volume change and outcome no clinically useful cut-off values could be selected.

At individual nodal level the analysis was done both in- and excluding completely responding lymph nodes. Including these lymph nodes yields a theoretical predictive prognostic effect of size change of pathological lymph nodes after treatment. Excluding them delivers us more clinically useful information about the predictive effect of the size change of a residual lymph node after treatment. In the clinic, disappeared pathological lymph nodes on post-treatment CT is considered obviously as a favorable prognostic sign. However, even after excluding disappearing pathological lymph nodes, the relationship between volume change and LNR remained significant. Our results are in concordance with the data published by Clavel et al. In patients with node-positive head and neck cancer, a significant effect of volume change after CRT was reported [16]. In the light of adaptive RT, volume change of tumor and pathological lymph nodes during treatment has been studied extensively. However, data on the prognostic value of volume change after but also during RT are scarce. Mishra et al. failed to find a correlation between nodal volume change during (C)RT and neck control in 38 patients with locoregionally advanced head and neck cancer [17]. Besides volume change, also the impact of change of largest nodal axial diameter on LNR was considered. Our data revealed, both in- and excluding completely responding pathological lymph nodes, a significant correlation between diameter change and outcome.

Because of this linear relationship no cut-off value could be selected. Clavel et al. described a negative predictive value (NPV) of 100% for a diameter decrease of > 80%. Hamilton et al. and Ojiri et al. proposed a cutoff value of >50% with NPV’s of 94 and 100% respectively [16, 18, 19]. An extensive discussion of the role of other imaging modalities of the neck after (C)RT such as ultrasound, PET-CT, and MRI is beyond the scope of this article, but each of those are believed to be useful in assessing response and the need for salvage neck dissection. Ultrasound (US), in combination with fine needle aspiration cytology (FNAC), is an inexpensive and readily available tool. Yom et al. described a NPV of 95% for US-FNAC in the non-irradiated neck [20, 21]. Diagnostic efficacy and in particular NPV of FNAC for residual or recurrent cervical lymph node metastasis is however significantly reduced after previous RT [22]. Data on the role of post-treatment PET-CT are emerging rapidly, and results are promising. Loo et al. described a NPV of 100% for PET-CT obtained 3 months after completion of radiotherapy [23]. This was further investigated in a multi-center study (PET-NECK), which recruited 564 patients with N2 or N3 head and neck cancer treated with CRT and randomized between routine ND and a wait and see approach if PET-CT 9-13 weeks after treatment shows no abnormal FDGuptake in the neck [24]. This study reports similar results in patients who underwent PET-CT-guided surveillance and those who underwent planned neck dissection, but surveillance resulted in fewer operations and was more cost-effective. In the last few years advances in MRI, with development of diffusion weighted MR (DW-MRI) and dynamic contrast-enhanced MRI, have provided additional information. Recently, Vandecaveye et al. evaluated response after RT in 29 patients with DW-MRI at three weeks after completion of treatment. He reported a NPV of 96% for adenopathies per neck side, and a sensitivity of 78% of DW-MRI versus 67% for conventional MRI for detecting sub centimeter lymph node metastasis [25].

This was a monocentric retrospective study, with its inherent limitations, analyzing a heterogeneous patient population over a large period of time. Nevertheless, the strength of this study lies in the large number of analyzed data and the fact that all measurements on the CT scans were performed by the same observer, with cooperation of both a radiation oncologist and radiologist specialized in head and neck cancer. Moreover, sufficient follow-up was carried out in a uniform manner.

## Conclusions

Our data show the prognostic value of both location as well as size change of pathological lymph nodes after (C)RT in locoregionally advanced head and neck cancer. Involvement of level VI-VII entails the highest metastatic risk. In the follow-up of patients with lymph node involvement of level VI-VII, the threshold to do radiological examinations should be lower.

The less decrease of lymph node volume and largest axial diameter after treatment, the worse the outcome. Patients with less decrease in volume and largest axial diameter as evaluated on the post-treatment CT scan might therefore benefit from a more closer radiological and clinical follow-up of the neck.

## Abbreviations

CRT: Chemo-radiotherapy
CT: Computed Tomography
DFS: Disease free survival
DW-MRI: Diffusion weighted MR
FNAC: Fine needle aspiration cytology
GEE: Generalized estimating equations
HPV: Human papilloma virus
HR: Hazard ratios
LC: Local control
LNR: Lymph node relapse
MFS: Metastasis free survival
NPV: Negative predictive value
OR: Odds ratios
OS: Overall survival
PCR: Polymerase chain reaction
RC: Regional control
RT: Radiation therapy
US: Ultrasound

## References

[1] Joseph A, D’Souza G (2012). Epidemiology of human papillomavirus-related head and neck cancer. Otolaryngol Clin N Am.

[2] Siegel R, Ma J, Zou Z (2014). Cancer statistics, 2014. CA Cancer J Clin.

[3] Pignon JP, Bourhis J, Domenge C (2000). Chemotherapy added to locoregional treatment for head and neck squamous-cell carcinoma: three meta-analysis of updated individual data. MACH-NC Collaborative Group. Meta-analysis of chemotherapy on head and neck cancer. Lancet.

[4] Wendt TG, Grabenbauer GG, Rödel CM (1998). Simultaneous radiochemotherapy versus radiotherapy alone in advanced head and neck cancer: a randomized multicenter study. J Clin Oncol.

[5] Nuyts S, Dirix P, Clement P (2009). Impact of adding concomitant chemotherapy to hyperfractionated accelerated radiotherapy for advanced head-and-neck squamous cell carcinoma. Int J Radiat Oncol Biol Phys.

[6] Nuyts S, Dirix P, Hermans R (2007). Early experience with a hybrid accelerated radiotherapy schedule for locally advanced head and neck cancer. Head Neck.

[7] Dawson LA, Anzai Y, Marsh L (2000). Patterns of local-regional recurrence following parotid-sparing conformal and segmental intensity-modulated radiotherapy for head and neck cancer. Int J Radiat Oncol Biol Phys.

[8] Denaro N, Russi EG, Numico G (2013). The role of neck dissection after radical chemoradiation for locally advanced head and neck cancer: should we move back?. Oncology.

[9] Liauw SL, Mancuso AA, Amdur RJ (2006). Postradiotherapy neck dissection for lymph node-positive head and neck cancer: the use of computed tomography to manage the neck. J Clin Oncol.

[10] Ojiri H, Mendenhall WM, Stringer SP (2002). Post-RT CT results as a predictive model for the necessity of planned post-RT neck dissection in patients with cervical metastatic disease from squamous cell carcinoma. Int J Radiat Oncol Biol Phys.

[11] Jones AS, Roland NJ, Field JK (1994). The level of cervical lymph node metastases: their prognostic relevance and relationship with head and neck squamous carcinoma primary sites. Clin Otolaryngol Allied Sci.

[12] McLaughlin MP, Mendenhall WM, Mancuso AA (1995). Retropharyngeal adenopathy as a predictor of outcome in squamous cell carcinoma of the head and neck. Head Neck.

[13] Gunn GB, Debnam JM, Fuller CD (2013). The impact of radiographic retropharyngeal adenopathy in oropharyngeal cancer. Cancer.

[14] Samuels S, Vainshtein J, Spector M (2015). Impact of retropharyngeal adenopathy on distant control and survival in HPV-related oropharyngeal cancer treated with chemoradiotherapy. Radiother Oncol.

[15] Dirix P, Nuyts S, Bussels B, Hermans R, Van den Bogaert W (2006). Prognostic influence of retropharyngeal lymph node metastasis in squamous cell carcinoma of the oropharynx. Int J Radiat Oncol Biol Phys.

[16] Clavel S, Charron MP, Bélair M (2012). The role of computed tomography in the management of the neck after chemoradiotherapy in patients with head and neck cancer. Int J Radiat Oncol Biol Phys.

[17] Mishra S, Hammond A, Read N (2013). Can radiological changes in lymph node volume during treatment predict success of radiation therapy in patients with locally advanced head and neck squamous cell carcinoma?. J Med Imaging Radiat Oncol.

[18] Hamilton JD, Ahmed S, Sandulache VC (2013). Improving diagnosis of persistent nodal metastases after definitive therapy for oropharyngeal carcinoma: specific signs for CT and best performance of combined criteria. Am J Neuroradiol.

[19] Ojiri H, Mancuso AA, Mendenhall WM (2002). Lymph nodes of patients with regional metastases from head and neck squamous cell carcinoma as a predictor of pathologic outcome: size changes at CT before and after radiation therapy. Am J Neuroradiol.

[20] Yom SS, Garden AS, Staerkel GA (2011). Sonographic examination of the neck after definitive radiotherapy for node-positive oropharyngeal cancer. Am J Neuroradiol.

[21] Furukawa MK, Furukawa M (2010). Diagnosis of lymph node metastases of head and neck cancer and evaluation of effects of chemoradiotherapy using ultrasonography. Int J Clin Oncol.

[22] Chan JY, Chan RC, Chow VL, To VS, Wei WI (2013). Efficacy of fine-needle aspiration in diagnosing cervical nodal metastasis from nasopharyngeal carcinoma after radiotherapy. Laryngoscope.

[23] Loo SW, Geropantas K, Beadsmoore C (2011). Neck dissection can be avoided after sequential chemoradiotherapy and negative post-treatment positron-emission tomography-computed tomography in N2 head and neck squamous cell carcinoma. Clin Oncol.

[24] Mehanna H, Wong W, McConkey C (2016). PET-CT survaillance versus neck dissection in advanced head and neck cancer. N Engl J Med.

[25] Vandecaveye V, Dirix P, De Keyzer F (2012). Diffusion-weighted magnetic resonance imaging early after chemoradiotherapy to monitor treatment response in head-and neck squamous cell carcinoma. Int J Radiat Oncol Biol Phys.

